# Supplementary material for: A Comparison of Depression and Anxiety among University Students in Nine Countries during the COVID-19 Pandemic
Source: J Clin Med. 2021 Jun 29;10(13):2882. doi: 10.3390/jcm10132882 (PMC8269122; doi:10.3390/jcm10132882)
Supplement: Supplementary file 1 [file jcm-10-02882-s001.zip › jcm-1209924-supplementary.pdf]

Supplementary Materials

Table S1. Association between depression risk and other variables among university students from Colombia, Czechia, Germany, Israel, Poland, Russia, Slovenia, Turkey, and Ukraine during the first wave of the COVID-19 pandemic

	Depression																	
	Colombia (n = 155)		Czechia (n = 310)		Germany (n = 270)		Israel (n = 199)		Poland (n = 301)		Russia (n = 285)		Slovenia (n = 209)		Turkey (n = 310)		Ukraine (n = 310)	
	No risk	Risk	No risk	Risk	No risk	Risk	No risk	Risk	No risk	Risk	No risk	Risk	No risk	Risk	No risk	Risk	No risk	Risk
Variable	n(%)	n(%)	n(%)	n(%)	n(%)	n(%)	n(%)	n(%)	n(%)	n(%)	n(%)	n(%)	n(%)	n(%)	n(%)	n(%)	n(%)	n(%)
Gender	χ^2(1) = 4.44*		χ^2(1) = 0.17		χ^2(1) = 0.18		χ^2(1) = 1.85		χ^2(1) = 7.28**		χ^2(1) = 10.24**		χ^2(1) = 0.96		χ^2(1) = 15.40***		χ^2(1) = 9.02**	
Men	32(62)	20(38)	81(78)	23(22)	45(60)	30(40)	33(66)	17(34)	51(64)	29(36)	67(73)	25(27)	23(74)	8(26)	66(50)	67(50)	77(83)	16(17)
Women	44(44)	57(56)	163(80)	41(20)	120(63)	71(37)	82(55)	67(45)	102(46)	119(54)	101(53)	90(47)	116(65)	62(35)	48(28)	125(72)	143(66)	74(34)
Place of Residence			χ^2(1) = 0.40		χ^2(1) = 0.92		χ^2(1) = 0.00		χ^2(1) = 0.49		χ^2(1) = 0.31		χ^2(1) = 0.32		χ^2(1) = 0.24		χ^2(1) = 0.91	
Village/Town	0(0)	0(0)	175(78)	49(22)	143(63)	84(37)	22(58)	16(42)	117(50)	118(50)	54(61)	34(39)	98(65)	52(35)	37(40)	56(60)	157(69)	69(31)
City	76(50)	77(50)	70(81)	16(19)	22(55)	18(45)	93(58)	68(42)	36(55)	30(45)	114(58)	83(42)	41(69)	18(31)	80(37)	137(63)	63(75)	21(25)
Level of Study	χ^2(1) = 4.16*		χ^2(1) = 5.71*		χ^2(1) = 0.04		χ^2(1) = 0.10		χ^2(1) = 0.83		χ^2(1) = 0.04		χ^2(1) = 8.24**		χ^2(1) = 1.36		χ^2(1) = 0.63	
Bachelor	78(52)	73(48)	171(76)	55(24)	82(61)	52(39)	113(58)	83(42)	83(49)	88(51)	145(59)	100(41)	86(60)	57(40)	104(37)	179(63)	205(70)	86(30)
Master	0(0)	4(100)	74(88)	10(12)	83(62)	50(38)	2(67)	1(33)	70(54)	60(46)	23(58)	17(42)	53(80)	13(20)	13(48)	14(52)	15(79)	4(21)
Exposure to COVID-19	χ^2(1) = 3.73*		χ^2(1) = 11.44***		χ^2(1) = 4.16*		χ^2(1) = 15.99***		χ^2(1) = 7.60**		χ^2(1) = 10.39**		χ^2(1) = 23.01***		χ^2(1) = 3.55		χ^2(1) = 6.65**	
Lower	17(68)	8(32)	155(86)	26(14)	69(70)	30(30)	50(78)	14(22)	56(63)	33(37)	56(75)	19(25)	67(87)	10(13)	29(48)	31(52)	53(84)	10(16)
Higher	61(47)	69(53)	90(70)	39(30)	96(57)	72(43)	65(48)	70(52)	96(45)	115(55)	112(53)	98(47)	72(55)	60(45)	88(35)	162(65)	167(68)	80(32)
Impact COVID-19: Total	χ^2(1) = 2.28		χ^2(1) = 16.10***		χ^2(1) = 39.80***		χ^2(1) = 35.64***		χ^2(1) = 20.85***		χ^2(1) = 19.72***		χ^2(1) = 33.96***		χ^2(1) = 9.26**		χ^2(1) = 13.01***	
Lower	21(62)	13(38)	158(87)	24(13)	124(77)	37(23)	87(76)	28(24)	57(73)	21(27)	108(71)	44(29)	102(82)	22(18)	39(53)	35(47)	118(81)	28(19)
Higher	57(47)	64(53)	87(68)	41(32)	41(39)	65(61)	28(33)	56(67)	96(43)	127(57)	60(45)	73(55)	37(44)	48(56)	78(33)	158(67)	102(62)	62(38)
Impact COVID-19: Graduation	χ^2(1) = 3.94*		χ^2(1) = 2.64		χ^2(1) = 16.95***		χ^2(1) = 12.94***		χ^2(1) = 3.43		χ^2(1) = 15.66***		χ^2(1) = 32.67***		χ^2(1) = 6.09*		χ^2(1) = 4.96*	
Lower	32(62)	20(38)	137(83)	29(17)	101(74)	36(26)	68(71)	28(29)	44(60)	29(40)	93(72)	37(28)	88(85)	15(15)	50(47)	56(53)	96(78)	27(22)
Higher	46(45)	57(55)	108(75)	36(25)	64(49)	66(51)	47(46)	56(54)	109(48)	119(52)	75(48)	80(52)	51(48)	55(52)	67(33)	137(67)	124(66)	63(34)
Impact COVID-19: Financing	χ^2(1) = 5.06*		χ^2(1) = 8.46**		χ^2(1) = 14.02***		χ^2(1) = 13.12***		χ^2(1) = 2.20		χ^2(1) = 11.25***		χ^2(1) = 10.10**		χ^2(1) = 13.58***		χ^2(1) = 4.65*	
Lower	29(64)	16(36)	125(86)	20(14)	117(70)	49(30)	58(73)	21(27)	49(58)	36(42)	71(72)	27(28)	76(78)	22(22)	59(51)	57(49)	95(78)	27(22)
Higher	49(45)	61(55)	120(73)	45(27)	48(48)	53(52)	57(48)	63(62)	104(48)	112(52)	97(52)	90(48)	63(57)	48(43)	58(30)	136(70)	125(66)	63(34)
Impact COVID-19: Relationships	χ^2(1) = 1.04		χ^2(1) = 11.06***		χ^2(1) = 9.76**		χ^2(1) = 14.38***		χ^2(1) = 24.57***		χ^2(1) = 24.16***		χ^2(1) = 13.70***		χ^2(1) = 4.26*		χ^2(1) = 8.36**	
Lower	16(59)	11(41)	147(86)	24(14)	89(72)	35(28)	71(71)	29(29)	66(73)	25(27)	114(72)	45(28)	91(77)	27(23)	21(53)	19(48)	118(79)	32(21)
Higher	62(48)	66(52)	98(71)	41(29)	76(53)	67(47)	44(44)	55(56)	87(41)	123(59)	54(43)	72(57)	48(53)	43(47)	96(36)	174(64)	102(64)	58(36)
Physical Activity	χ^2(1) = 0.56		χ^2(1) = 1.70		χ^2(1) = 0.03		χ^2(1) = 3.88*		χ^2(1) = 4.85*		χ^2(1) = 7.36**		χ^2(1) = 0.44		χ^2(1) = 1.01		χ^2(1) = 11.77***	
Sufficient	44(48)	48(52)	168(81)	39(19)	79(62)	48(38)	65(52)	59(48)	90(46)	105(54)	102(53)	89(47)	51(61)	29(36)	97(37)	168(63)	107(63)	63(37)
Insufficient	34(54)	29(46)	77(75)	26(25)	85(61)	54(39)	50(67)	25(33)	63(59)	43(41)	66(70)	28(30)	88(68)	41(32)	20(44)	25(56)	113(81)	27(19)
Physical Health	χ^2(1) = 5.14*		χ^2(1) = 3.44		χ^2(1) = 21.71***		χ^2(1) = 4.88*		χ^2(1) = 13.14***		χ^2(1) = 9.99**		χ^2(1) = 15.16***		χ^2(1) = 5.89*		χ^2(1) = 3.60	
Better	75(53)	66(47)	236(80)	59(20)	153(68)	73(32)	113(59)	77(41)	150(54)	129(46)	148(63)	86(37)	134(71)	56(29)	107(41)	157(59)	215(72)	84(28)
Worse	3(21)	11(79)	9(60)	6(40)	12(29)	29(71)	2(22)	7(78)	3(14)	19(86)	20(39)	31(61)	5(26)	14(74)	10(22)	36(78)	5(45)	6(55)
Physical Health Comparative	χ^2(1) = 6.83**		χ^2(1) = 14.45***		χ^2(1) = 7.09**		χ^2(1) = 5.76*		χ^2(1) = 21.64***		χ^2(1) = 2.33		χ^2(1) = 20.89***		χ^2(1) = 10.73		χ^2(1) = 6.96**	
Better	73(54)	61(46)	228(82)	50(18)	139(66)	72(34)	108(61)	70(39)	146(56)	114(44)	135(61)	85(39)	131(72)	50(28)	111(41)	158(59)	215(72)	82(28)
Worse	5(24)	16(76)	17(53)	15(47)	26(46)	30(54)	7(33)	14(67)	7(17)	34(83)	33(51)	32(49)	8(29)	20(71)	6(15)	35(85)	5(38)	8(62)

Note. PIC = Perceived Impact of COVID-19 on Students' Well-being; PH = Physical Health. **p* < 0.05, ***p* < 0.01, ****p* < 0.001

Table S2. Association between anxiety risk and other variables among university students from Colombia, Czechia, Germany, Israel, Poland, Russia, Slovenia, Turkey, and Ukraine during the first wave of the COVID-19 pandemic

	Anxiety																	
	Colombia (n = 155)		Czechia (n = 310)		Germany (n = 270)		Israel (n = 199)		Poland (n = 301)		Russia (n = 285)		Slovenia (n = 209)		Turkey (n = 310)		Ukraine (n = 310)	
	No risk	Risk	No risk	Risk	No risk	Risk	No risk	Risk	No risk	Risk	No risk	Risk	No risk	Risk	No risk	Risk	No risk	Risk
Variable	n(%)	n(%)	n(%)	n(%)	n(%)	n(%)	n(%)	n(%)	n(%)	n(%)	n(%)	n(%)	n(%)	n(%)	n(%)	n(%)	n(%)	n(%)
Gender	$\chi^2(1) = 3.55$		$\chi^2(1) = 3.55$		$\chi^2(1) = 1.37$		$\chi^2(1) = 4.87$*		$\chi^2(1) = 3.82$		$\chi^2(1) = 4.15$*		$\chi^2(1) = 2.45$		$\chi^2(1) = 9.15$**		$\chi^2(1) = 7.52$**	
Men	37(71)	15(29)	91(88)	13(12)	73(97)	26(84)	5(16)	10(20)	50(63)	30(38)	69(75)	23(25)	26(84)	5(16)	77(58)	56(42)	81(87)	12(13)
Women	56(55)	45(45)	177(87)	27(13)	181(94)	125(70)	53(30)	55(37)	110(50)	111(50)	120(63)	71(37)	125(70)	53(30)	70(40)	103(60)	158(73)	59(27)
Place of Residence			$\chi^2(1) = 1.21$		$\chi^2(1) = 0.57$		$\chi^2(1) = 1.72$		$\chi^2(1) = 7.66$**		$\chi^2(1) = 0.51$		$\chi^2(1) = 2.25$		$\chi^2(1) = 0.67$		$\chi^2(1) = 0.46$	
Village/Town	0(0)	0(0)	198(88)	26(12)	219(95)	104(69)	46(31)	9(24)	115(49)	120(51)	61(69)	27(31)	104(69)	46(31)	42(45)	51(55)	172(76)	54(24)
City	95(61)	60(39)	72(84)	14(16)	37(93)	47(80)	12(20)	56(35)	45(68)	21(32)	128(65)	69(35)	47(80)	12(20)	109(50)	108(50)	67(80)	17(20)
Level of Study	$\chi 2(1) = 2.27$		$\chi 2(1) = 2.27$		$\chi^2(1) = 0.01$		$\chi^2(1) = 1.48$		$\chi^2(1) = 0.20$		$\chi^2(1) = 0.83$		$\chi^2(1) = 5.91$*		$\chi^2(1) = 0.55$		$\chi^2(1) = 0.58$	
Bachelor	94(62)	57(38)	190(84)	36(16)	130(95)	96(67)	47(33)	65(33)	89(52)	82(48)	165(67)	80(33)	96(67)	47(33)	136(48)	147(52)	223(77)	68(23)
Master	1(25)	3(75)	80(95)	4(5)	126(95)	55(83)	11(17)	0(0)	71(55)	59(45)	24(60)	16(40)	55(83)	11(17)	15(56)	12(44)	16(84)	3(16)
Exposure to COVID-19	$\chi^2(1) = 4.40$*		$\chi^2(1) = 4.40$*		$\chi^2(1) = 8.82$**		$\chi^2(1) = 10.27$**		$\chi^2(1) = 8.97$**		$\chi^2(1) = 6.95$		$\chi^2(1) = 13.25$***		$\chi^2(1) = 7.90$		$\chi^2(1) = 10.03$**	
Lower	20(80)	5(20)	167(92)	14(8)	101(100)	67(87)	10(13)	11(17)	59(66)	30(34)	59(79)	16(21)	67(87)	10(13)	39(65)	21(35)	58(92)	5(8)
Higher	75(58)	55(42)	103(80)	26(20)	155(92)	84(64)	48(36)	54(40)	100(47)	111(53)	130(62)	80(38)	84(64)	48(36)	112(45)	138(55)	181(73)	66(27)
PIC: Total	$\chi^2(1) = 8.14$**		$\chi^2(1) = 8.14$**		$\chi^2(1) = 9.36$**		$\chi^2(1) = 32.27$***		$\chi^2(1) = 12.73$***		$\chi^2(1) = 5.34$*		$\chi^2(1) = 29.98$***		$\chi^2(1) = 11.92$***		$\chi^2(1) = 17.48$***	
Lower	28(82)	6(18)	169(93)	13(7)	160(98)	107(86)	17(14)	19(17)	55(71)	23(29)	110(72)	42(28)	107(86)	17(14)	49(66)	25(34)	128(88)	18(12)
Higher	67(55)	54(45)	101(79)	27(21)	96(90)	44(52)	41(48)	46(55)	105(47)	118(53)	79(59)	54(41)	44(52)	41(48)	102(43)	134(57)	111(68)	53(32)
PIC: Qualifications	$\chi^2(1) = 8.06$**		$\chi^2(1) = 8.06$**		$\chi^2(1) = 11.62$***		$\chi^2(1) = 9.81$**		$\chi^2(1) = 3.76$		$\chi^2(1) = 7.37$**		$\chi^2(1) = 23.19$***		$\chi^2(1) = 8.78$**		$\chi^2(1) = 11.31$***	
Lower	40(77)	12(23)	157(95)	9(5)	138(99)	90(87)	13(13)	21(22)	46(63)	27(37)	97(75)	33(25)	90(87)	13(13)	64(60)	42(40)	107(87)	16(13)
Higher	55(53)	48(47)	113(78)	31(22)	118(90)	61(58)	45(42)	44(43)	114(50)	114(50)	92(59)	63(41)	61(58)	45(42)	87(43)	117(57)	132(71)	55(29)
PIC: Economic Status	$\chi^2(1) = 1.54$		$\chi^2(1) = 1.54$		$\chi^2(1) = 4.28$*		$\chi^2(1) = 4.42$*		$\chi^2(1) = 0.52$		$\chi^2(1) = 7.47$*		$\chi^2(1) = 4.96$*		$\chi^2(1) = 15.00$***		$\chi^2(1) = 3.69$	
Lower	31(69)	14(31)	138(95)	7(5)	162(97)	78(80)	20(20)	19(24)	48(56)	37(44)	73(74)	25(26)	78(80)	20(20)	73(63)	43(37)	101(83)	21(17)
Higher	64(58)	46(42)	132(80)	33(20)	94(91)	73(66)	38(34)	46(38)	112(52)	104(48)	116(62)	71(38)	73(66)	38(34)	78(40)	116(60)	138(73)	50(27)
PIC: Relationships	$\chi^2(1) = 5.62$*		$\chi^2(1) = 5.62$*		$\chi^2(1) = 0.07$		$\chi^2(1) = 14.66$***		$\chi^2(1) = 21.95$***		$\chi^2(1) = 10.04$**		$\chi^2(1) = 13.39$***		$\chi^2(1) = 4.88$*		$\chi^2(1) = 13.05$***	
Lower	22(81)	5(19)	155(91)	16(9)	119(95)	97(82)	21(18)	20(20)	67(74)	24(26)	118(74)	41(26)	97(82)	21(18)	26(65)	14(35)	129(86)	21(14)
Higher	73(57)	55(43)	115(83)	24(17)	137(94)	54(59)	37(41)	45(45)	93(44)	117(56)	71(56)	55(44)	54(59)	37(41)	125(46)	145(54)	110(69)	50(31)
Physical Activity	$\chi^2(1) = 0.64$		$\chi^2(1) = 0.64$		$\chi^2(1) = 3.21$		$\chi^2(1) = 2.94$		$\chi^2(1) = 7.94$**		$\chi^2(1) = 0.95$		$\chi^2(1) = 0.49$		$\chi^2(1) = 1.73$		$\chi^2(1) = 4.80$*	
Insufficient	54(59)	38(38)	176(85)	31(15)	119(92)	60(75)	20(25)	46(37)	92(47)	103(53)	123(64)	68(36)	60(75)	20(25)	125(47)	140(53)	123(72)	47(28)
Sufficient	41(65)	22(35)	94(91)	9(9)	135(97)	91(71)	38(29)	19(25)	68(64)	38(36)	66(70)	28(30)	91(71)	38(29)	26(58)	19(42)	116(83)	24(17)
PH	$\chi^2(1) = 4.24$*		$\chi^2(1) = 4.24$*		$\chi^2(1) = 8.38$**		$\chi^2(1) = 2.25$		$\chi^2(1) = 18.51$***		$\chi^2(1) = 4.97$*		$\chi^2(1) = 6.45$*		$\chi^2(1) = 13.29$***		$\chi^2(1) = 6.47$*	
Better	90(64)	51(36)	259(88)	36(12)	220(96)	142(75)	48(25)	60(32)	158(57)	121(43)	162(69)	72(31)	142(75)	48(25)	140(53)	124(47)	234(78)	65(22)
Worse	5(36)	9(64)	11(73)	4(27)	36(86)	9(47)	10(53)	5(56)	2(9)	20(91)	27(53)	24(47)	9(47)	10(53)	11(24)	35(76)	5(45)	6(55)
PH Comparative	$\chi^2(1) = 3.83$		$\chi^2(1) = 3.83$		$\chi^2(1) = 4.19$*		$\chi^2(1) = 2.39$		$\chi^2(1) = 18.56$***		$\chi^2(1) = 4.50$*		$\chi^2(1) = 10.75$**		$\chi^2(1) = 11.18$***		$\chi^2(1) = 1.86$	
Better	88(66)	46(34)	246(88)	32(12)	205(96)	138(76)	43(24)	55(31)	151(58)	109(42)	153(70)	67(30)	138(76)	43(24)	141(52)	128(48)	231(78)	66(22)
Worse	7(33)	14(67)	24(75)	8(25)	51(89)	13(46)	15(54)	10(48)	9(22)	32(78)	36(55)	29(45)	13(46)	15(54)	10(24)	31(76)	8(62)	5(38)

Note. PIC = Perceived Impact of COVID-19 on Students' Well-being; PH = Physical Health. *$p < 0.05$, **$p < 0.01$, ***$p < 0.001$

Table S3. Logistic regression for depression symptoms among university students from Colombia during the first wave of the COVID-19 pandemic

		95% *CI*					Wald Test			BCa 95% *CI*	
**Variable**	**Estimate**	**Lower bound**	**Upper bound**	**Standard Error**	**Odds Ratio**	*z*	**Wald Statistic**	*df*	*p*	**Lower bound**	**Upper bound**
Intercept	-1.809	-3.133	-0.485	0.676	0.164	-2.678	7.171	1	0.007	-3.35	-0.324
Gender (Women)	0.760	0.004	1.516	0.386	2.138	1.970	3.883	1	0.049	-0.092	1.539
Place of Residence (City)	-	-	-	-	-	-	-	-	-	-	-
Level of Study (Master)	-	-	-	-	-	-	-	-	-	-	-
Exposure to COVID-19 (High)	0.654	-0.364	1.672	0.519	1.923	1.260	1.587	1	0.208	-0.579	1.917
PIC Total (High)	-0.356	-1.631	0.919	0.650	0.700	-0.548	0.300	1	0.584	-1.812	1.200
PIC Qualifications (High)	0.206	-0.710	1.123	0.468	1.229	0.441	0.195	1	0.659	-0.85	1.223
PIC Economic Status (High)	0.486	-0.343	1.316	0.423	1.626	1.149	1.320	1	0.251	-0.519	1.350
PIC Relationships (High)	0.463	-0.680	1.605	0.583	1.589	0.794	0.630	1	0.427	-0.996	1.847
Physical Activity (Insufficient)	-0.138	-0.853	0.577	0.365	0.871	-0.379	0.144	1	0.705	-0.938	0.627
Physical Health (Worse)	1.242	-0.453	2.937	0.865	3.462	1.436	2.063	1	0.151	-1.219	17.191
Physical Health Comparative (Worse)	1.175	-0.111	2.460	0.656	3.237	1.791	3.208	1	0.073	-0.546	2.598

Note. *CI* = confidence interval; BCa = Bias-corrected accelerated. Bootstrapping based on 5,000 successful replicates.

Table S4. Logistic regression for depression symptoms among university students from Czechia during the first wave of the COVID-19 pandemic

		95% *CI*					Wald Test			BCa 95% *CI*	
**Variable**	**Estimate**	**Lower bound**	**Upper bound**	**Standard Error**	**Odds Ratio**	*z*	**Wald Statistic**	*df*	*p*	**Lower bound**	**Upper bound**
Intercept	-2.382	-3.227	-1.536	0.431	0.092	-5.520	30.474	1	< .001	-3.454	-1.345
Gender (Women)	-0.026	-0.667	0.615	0.327	0.974	-0.079	0.006	1	0.937	-0.687	0.779
Place of Residence (City)	-0.173	-0.880	0.534	0.361	0.841	-0.479	0.230	1	0.632	-0.943	0.623
Level of Study (Master)	-0.546	-1.339	0.248	0.405	0.579	-1.348	1.818	1	0.178	-1.402	0.350
Exposure to COVID-19 (High)	0.659	0.052	1.267	0.310	1.933	2.128	4.526	1	0.033	-0.017	1.275
PIC Total (High)	0.821	-0.139	1.781	0.490	2.273	1.677	2.813	1	0.094	-0.400	1.901
PIC Qualifications (High)	-0.323	-1.099	0.452	0.396	0.724	-0.817	0.668	1	0.414	-1.274	0.632
PIC Economic Status (High)	0.444	-0.279	1.166	0.369	1.559	1.204	1.449	1	0.229	-0.402	1.275
PIC Relationships (High)	0.210	-0.553	0.974	0.389	1.234	0.540	0.292	1	0.589	-0.673	1.034
Physical Activity (Insufficient)	0.366	-0.253	0.984	0.315	1.442	1.159	1.344	1	0.246	-0.335	0.988
Physical Health (Worse)	-0.117	-1.481	1.247	0.696	0.890	-0.168	0.028	1	0.866	-2.116	1.570
Physical Health Comparative (Worse)	1.301	0.396	2.207	0.462	3.673	2.816	7.930	1	0.005	0.119	2.223

Note. *CI* = confidence interval; BCa = Bias-corrected accelerated. Bootstrapping based on 5,000 successful replicates.

Table S5. Logistic regression for depression symptoms among university students from Germany during the first wave of the COVID-19 pandemic

Variable	Estimate	95% *CI*		Standard Error	Odds Ratio	*z*	Wald Test			BCa 95% *CI*	
		Lower bound	Upper bound				Wald Statistic	*df*	*p*	Lower bound	Upper bound
Intercept	-2.036	-2.978	-1.095	0.480	0.131	-4.239	17.97	1	< .001	-2.967	-1.035
Gender (Women)	-0.138	-0.775	0.499	0.325	0.871	-0.426	0.181	1	0.670	-0.816	0.608
Place of Residence (City)	0.359	-0.445	1.162	0.410	1.431	0.875	0.765	1	0.382	-0.596	1.237
Level of Study (Master)	-0.073	-0.643	0.498	0.291	0.930	-0.250	0.063	1	0.802	-0.699	0.564
Exposure to COVID-19 (High)	0.337	-0.271	0.945	0.310	1.401	1.086	1.179	1	0.278	-0.319	0.982
PIC Total (High)	1.326	0.483	2.170	0.430	3.768	3.083	9.505	1	0.002	0.351	2.101
PIC Qualifications (High)	0.274	-0.439	0.988	0.364	1.315	0.753	0.567	1	0.451	-0.531	0.992
PIC Economic Status (High)	0.261	-0.387	0.909	0.331	1.298	0.789	0.623	1	0.430	-0.431	0.934
PIC Relationships (High)	0.197	-0.475	0.869	0.343	1.218	0.574	0.330	1	0.566	-0.557	0.903
Physical Activity (Insufficient)	0.441	-0.165	1.048	0.310	1.555	1.426	2.033	1	0.154	-0.255	1.068
Physical Health (Worse)	1.382	0.419	2.345	0.491	3.983	2.812	7.909	1	0.005	0.232	2.395
Physical Health Comparative (Worse)	0.226	-0.619	1.071	0.431	1.253	0.523	0.274	1	0.601	-0.728	1.150

Note. CI = confidence interval; BCa = Bias-corrected accelerated. Bootstrapping based on 5,000 successful replicates.

Table S6. Logistic regression for depression symptoms among university students from Israel during the first wave of the COVID-19 pandemic

		95% *CI*					Wald Test			BCa 95% *CI*	
**Variable**	**Estimate**	**Lower bound**	**Upper bound**	**Standard Error**	**Odds Ratio**	*z*	**Wald Statistic**	*df*	*p*	**Lower bound**	**Upper bound**
Intercept	-1.911	-3.078	-0.745	0.595	0.148	-3.212	10.314	1	0.001	-3.116	-0.589
Gender (Women)	0.515	-0.268	1.297	0.399	1.673	1.289	1.661	1	0.198	-0.366	1.307
Place of Residence (City)	-0.379	-1.229	0.470	0.433	0.684	-0.875	0.766	1	0.381	-1.368	0.597
Level of Study (Master)	-	-	-	-	-	-	-	-	-	-	-
Exposure to COVID-19 (High)	0.862	0.092	1.633	0.393	2.368	2.193	4.809	1	0.028	-0.059	1.700
PIC Total (High)	1.348	0.302	2.394	0.534	3.851	2.527	6.385	1	0.012	0.147	2.524
PIC Qualifications (High)	-0.125	-1.026	0.777	0.460	0.883	-0.271	0.073	1	0.786	-1.196	0.889
PIC Economic Status (High)	0.227	-0.611	1.065	0.428	1.255	0.532	0.283	1	0.595	-0.732	1.167
PIC Relationships (High)	0.468	-0.279	1.215	0.381	1.596	1.227	1.505	1	0.220	-0.338	1.271
Physical Activity (Insufficient)	-0.290	-0.980	0.400	0.352	0.748	-0.824	0.679	1	0.410	-1.037	0.475
Physical Health (Worse)	-	-	-	-	-	-	-	-	-	-	-
Physical Health Comparative (Worse)	0.792	-0.300	1.884	0.557	2.207	1.421	2.019	1	0.155	-0.418	1.936

Note. *CI* = confidence interval; BCa = Bias-corrected accelerated. Bootstrapping based on 5,000 successful replicates.

Table S7. Logistic regression for depression symptoms among university students from Poland during the first wave of the COVID-19 pandemic

		95% *CI*					Wald Test			BCa 95% *CI*	
**Variable**	**Estimate**	**Lower bound**	**Upper bound**	**Standard Error**	**Odds Ratio**	*z*	**Wald Statistic**	*df*	*p*	**Lower bound**	**Upper bound**
Intercept	-2.045	-3.034	-1.056	0.505	0.129	-4.052	16.419	1	< .001	-2.964	-0.942
Gender (Women)	0.784	0.190	1.377	0.303	2.190	2.589	6.701	1	0.010	0.115	1.393
Place of Residence (City)	0.047	-0.55	0.645	0.305	1.049	0.156	0.024	1	0.876	-0.598	0.703
Level of Study (Master)	-0.169	-0.698	0.360	0.270	0.845	-0.625	0.390	1	0.532	-0.733	0.436
Exposure to COVID-19 (High)	0.548	-0.043	1.138	0.301	1.729	1.818	3.305	1	0.069	-0.079	1.148
PIC Total (High)	0.517	-0.396	1.431	0.466	1.678	1.110	1.232	1	0.267	-0.488	1.560
PIC Qualifications (High)	-0.138	-0.871	0.594	0.374	0.871	-0.370	0.137	1	0.711	-0.942	0.677
PIC Economic Status (High)	0.005	-0.63	0.640	0.324	1.005	0.016	2.604e -4	1	0.987	-0.644	0.731
PIC Relationships (High)	1.000	0.299	1.700	0.358	2.718	2.796	7.819	1	0.005	0.197	1.653
Physical Activity (Insufficient)	-0.281	-0.822	0.261	0.276	0.755	-1.016	1.032	1	0.310	-0.878	0.280
Physical Health (Worse)	1.187	-0.318	2.691	0.767	3.276	1.546	2.391	1	0.122	-1.032	3.447
Physical Health Comparative (Worse)	1.268	0.286	2.250	0.501	3.554	2.531	6.408	1	0.011	0.126	2.323

Note. CI = confidence interval; BCa = Bias-corrected accelerated. Bootstrapping based on 5,000 successful replicates.

Table S8. Logistic regression for depression symptoms among university students from Russia during the first wave of the COVID-19 pandemic

Variable	Estimate	95% *CI*		Standard Error	Odds Ratio	*z*	Wald Test			BCa 95% *CI*	
		Lower bound	Upper bound				Wald Statistic	*df*	*p*	Lower bound	Upper bound
Intercept	-2.790	-3.826	-1.755	0.528	0.061	-5.281	27.889	1	< .001	-4.014	-1.579
Gender (Women)	0.930	0.300	1.560	0.321	2.534	2.893	8.367	1	0.004	0.209	1.590
Place of Residence (City)	-0.097	-0.697	0.503	0.306	0.908	-0.317	0.101	1	0.751	-0.767	0.606
Level of Study (Master)	0.539	-0.268	1.346	0.412	1.714	1.309	1.713	1	0.191	-0.343	1.384
Exposure to COVID-19 (High)	0.762	0.080	1.443	0.348	2.142	2.190	4.798	1	0.028	-0.018	1.535
PIC Total (High)	-0.482	-1.373	0.41	0.455	0.618	-1.059	1.122	1	0.290	-1.507	0.544
PIC Qualifications (High)	0.900	0.175	1.624	0.370	2.459	2.433	5.920	1	0.015	0.102	1.636
PIC Economic Status (High)	0.412	-0.267	1.091	0.347	1.510	1.188	1.412	1	0.235	-0.382	1.088
PIC Relationships (High)	1.39	0.681	2.100	0.362	4.016	3.842	14.758	1	< .001	0.495	2.157
Physical Activity (Insufficient)	-0.775	-1.375	-0.176	0.306	0.461	-2.535	6.425	1	0.011	-1.375	-0.137
Physical Health (Worse)	1.388	0.472	2.304	0.467	4.006	2.971	8.826	1	0.003	0.425	2.305
Physical Health Comparative (Worse)	-0.178	-1.004	0.647	0.421	0.837	-0.424	0.179	1	0.672	-1.029	0.683

Note. *CI* = confidence interval; BCa = Bias-corrected accelerated. Bootstrapping based on 5,000 successful replicates.

Table S9. Logistic regression for depression symptoms among university students from Slovenia during the first wave of the COVID-19 pandemic

Variable	Estimate	95% *CI*		Standard Error	Odds Ratio	*z*	Wald Test			BCa 95% *CI*	
		Lower bound	Upper bound				Wald Statistic	*df*	*p*	Lower bound	Upper bound
Intercept	-2.815	-4.221	-1.409	0.717	0.060	-3.925	15.402	1	< .001	-4.469	-1.061
Gender (Women)	-0.114	-1.193	0.966	0.551	0.893	-0.206	0.043	1	0.837	-1.736	1.353
Place of Residence (City)	-0.080	-0.911	0.752	0.424	0.923	-0.188	0.035	1	0.851	-1.105	0.885
Level of Study (Master)	-0.648	-1.481	0.186	0.425	0.523	-1.524	2.321	1	0.128	-1.618	0.313
Exposure to COVID-19 (High)	1.139	0.258	2.021	0.450	3.125	2.534	6.423	1	0.011	0.058	2.114
PIC Total (High)	0.661	-0.482	1.805	0.583	1.937	1.133	1.284	1	0.257	-0.773	1.999
PIC Qualifications (High)	1.260	0.337	2.183	0.471	3.524	2.675	7.154	1	0.007	0.155	2.076
PIC Economic Status (High)	0.131	-0.749	1.010	0.449	1.140	0.291	0.085	1	0.771	-0.834	1.129
PIC Relationships (High)	0.363	-0.575	1.302	0.479	1.438	0.759	0.576	1	0.448	-0.802	1.466
Physical Activity (Insufficient)	-0.074	-0.828	0.680	0.385	0.929	-0.192	0.037	1	0.848	-0.896	0.757
Physical Health (Worse)	1.053	-0.377	2.482	0.729	2.865	1.443	2.084	1	0.149	-0.669	2.539
Physical Health Comparative (Worse)	1.613	0.402	2.825	0.618	5.019	2.610	6.812	1	0.009	0.165	2.916

Note. *CI* = confidence interval; BCa = Bias-corrected accelerated. Bootstrapping based on 5,000 successful replicates.

Table S10. Logistic regression for depression symptoms among university students from Turkey during the first wave of the COVID-19 pandemic

		95% *CI*					Wald Test			BCa 95% *CI*	
**Variable**	**Estimate**	**Lower bound**	**Upper bound**	**Standard Error**	**Odds Ratio**	*z*	**Wald Statistic**	*df*	*p*	**Lower bound**	**Upper bound**
Intercept	-1.464	-2.568	-0.360	0.563	0.231	-2.599	6.752	1	0.009	-2.605	-0.249
Gender (Women)	0.906	0.398	1.413	0.259	2.474	3.499	12.241	1	< .001	0.350	1.429
Place of Residence (City)	0.275	-0.289	0.839	0.288	1.317	0.955	0.913	1	0.339	-0.335	0.88
Level of Study (Master)	-0.503	-1.370	0.363	0.442	0.604	-1.139	1.296	1	0.255	-1.367	0.345
Exposure to COVID-19 (High)	0.450	-0.187	1.087	0.325	1.568	1.384	1.916	1	0.166	-0.275	1.123
PIC Total (High)	0.115	-0.762	0.992	0.447	1.122	0.257	0.066	1	0.797	-0.840	1.066
PIC Qualifications (High)	0.116	-0.530	0.762	0.330	1.123	0.351	0.123	1	0.725	-0.654	0.808
PIC Economic Status (High)	0.606	-0.001	1.213	0.310	1.833	1.958	3.835	1	0.050	-0.084	1.246
PIC Relationships (High)	0.445	-0.422	1.312	0.442	1.561	1.007	1.013	1	0.314	-0.544	1.353
Physical Activity (Insufficient)	-0.248	-0.946	0.451	0.356	0.781	-0.695	0.483	1	0.487	-0.970	0.474
Physical Health (Worse)	-0.192	-1.279	0.894	0.554	0.825	-0.347	0.120	1	0.728	-1.622	1.134
Physical Health Comparative (Worse)	1.336	0.090	2.583	0.636	3.805	2.101	4.416	1	0.036	-0.283	2.602

Note. *CI* = confidence interval; BCa = Bias-corrected accelerated. Bootstrapping based on 5,000 successful replicates.

Table S11. Logistic regression for depression symptoms among university students from Ukraine during the first wave of the COVID-19 pandemic

		95% *CI*					Wald Test			BCa 95% *CI*	
**Variable**	**Estimate**	**Lower bound**	**Upper bound**	**Standard Error**	**Odds Ratio**	*z*	**Wald Statistic**	*df*	*p*	**Lower bound**	**Upper bound**
Intercept	-2.698	-3.772	-1.625	0.547	0.067	-4.929	24.293	1	< .001	-3.933	-1.322
Gender (Women)	0.803	0.150	1.456	0.333	2.231	2.409	5.806	1	0.016	0.070	1.512
Place of Residence (City)	-0.179	-0.800	0.442	0.317	0.836	-0.566	0.320	1	0.571	-0.864	0.496
Level of Study (Master)	-0.080	-1.317	1.156	0.631	0.923	-0.127	0.016	1	0.899	-1.698	1.337
Exposure to COVID-19 (High)	0.873	0.060	1.686	0.415	2.394	2.105	4.403	1	0.035	-0.037	1.749
PIC Total (High)	0.428	-0.420	1.276	0.433	1.534	0.989	0.978	1	0.323	-0.440	1.371
PIC Qualifications (High)	0.096	-0.608	0.800	0.359	1.101	0.268	0.072	1	0.789	-0.681	0.795
PIC Economic Status (High)	0.360	-0.282	1.001	0.327	1.433	1.099	1.208	1	0.272	-0.347	1.033
PIC Relationships (High)	0.479	-0.218	1.175	0.355	1.614	1.346	1.813	1	0.178	-0.281	1.230
Physical Activity (Insufficient)	-0.851	-1.413	-0.288	0.287	0.427	-2.965	8.791	1	0.003	-1.396	-0.218
Physical Health (Worse)	0.303	-1.149	1.755	0.741	1.354	0.409	0.168	1	0.682	-1.987	2.138
Physical Health Comparative (Worse)	1.211	-0.175	2.597	0.707	3.358	1.713	2.934	1	0.087	-0.798	2.750

Note. CI = confidence interval; BCa = Bias-corrected accelerated. Bootstrapping based on 5,000 successful replicates.

Table S12. Logistic regression for anxiety symptoms among university students from Colombia during the first wave of the COVID-19 pandemic

		95% *CI*					Wald Test			BCa 95% *CI*	
**Variable**	**Estimate**	**Lower bound**	**Upper bound**	**Standard Error**	**Odds Ratio**	*z*	**Wald Statistic**	*df*	*p*	**Lower bound**	**Upper bound**
Intercept	-3.041	-4.628	-1.455	0.810	0.048	-3.757	14.115	1	< .001	-4.956	-1.056
Gender (Women)	0.679	-0.126	1.484	0.411	1.971	1.652	2.730	1	0.098	-0.202	1.563
Place of Residence (City)	-	-	-	-	-	-	-	-	-	-	-
Level of Study (Master)	-	-	-	-	-	-	-	-	-	-	-
Exposure to COVID-19 (High)	0.916	-0.228	2.06	0.583	2.499	1.507	2.465	1	0.116	-0.497	2.229
PIC Total (High)	0.329	-1.058	1.716	0.708	1.389	0.464	0.216	1	0.642	-1.401	1.877
PIC Qualifications (High)	0.439	-0.527	1.405	0.493	1.551	0.890	0.793	1	0.373	-0.696	1.624
PIC Economic Status (High)	-0.169	-1.038	0.700	0.443	0.844	-0.382	0.146	1	0.703	-1.121	0.891
PIC Relationships (High)	0.857	-0.449	2.162	0.666	2.355	1.286	1.653	1	0.199	-0.743	2.474
Physical Activity (Insufficient)	-0.151	-0.903	0.601	0.383	0.860	-0.394	0.155	1	0.694	-0.948	0.706
Physical Health (Worse)	0.475	-0.947	1.897	0.726	1.609	0.655	0.429	1	0.512	-1.696	2.003
Physical Health Comparative (Worse)	1.346	0.132	2.560	0.619	3.843	2.173	4.723	1	0.030	-0.266	2.819

Note. *CI* = confidence interval; BCa = Bias-corrected accelerated. Bootstrapping based on 5,000 successful replicates.

Table S13. Logistic regression for anxiety symptoms among university students from Czechia during the first wave of the COVID-19 pandemic

		95% *CI*					Wald Test			BCa 95% *CI*	
**Variable**	**Estimate**	**Lower bound**	**Upper bound**	**Standard Error**	**Odds Ratio**	**z**	**Wald Statistic**	**df**	**p**	**Lower bound**	**Upper bound**
Intercept	-4.064	-5.334	-2.794	0.648	0.017	-6.272	39.338	1	< .001	-5.990	-2.250
Gender (Women)	0.369	-0.462	1.200	0.424	1.446	0.870	0.757	1	0.384	-0.589	1.542
Place of Residence (City)	0.788	-0.033	1.609	0.419	2.198	1.881	3.536	1	0.06	-0.257	1.677
Level of Study (Master)	-1.287	-2.476	-0.099	0.607	0.276	-2.122	4.505	1	0.034	-2.414	-0.060
Exposure to COVID-19 (High)	0.905	0.134	1.677	0.394	2.473	2.300	5.289	1	0.021	0.024	1.764
PIC Total (High)	-0.082	-1.313	1.149	0.628	0.921	-0.131	0.017	1	0.896	-1.472	1.254
PIC Qualifications (High)	1.185	0.180	2.190	0.513	3.272	2.312	5.345	1	0.021	-0.038	2.361
PIC Economic Status (High)	1.251	0.225	2.277	0.523	3.495	2.391	5.716	1	0.017	0.100	2.533
PIC Relationships (High)	0.094	-0.899	1.087	0.507	1.099	0.186	0.034	1	0.853	-1.029	1.223
Physical Activity (Insufficient)	-0.936	-1.832	-0.041	0.457	0.392	-2.049	4.200	1	0.040	-1.840	-0.037
Physical Health (Worse)	-0.546	-2.033	0.942	0.759	0.579	-0.719	0.517	1	0.472	-2.767	1.538
Physical Health Comparative (Worse)	1.120	0.006	2.234	0.568	3.064	1.970	3.881	1	0.049	-0.519	2.504

Note. *CI* = confidence interval; BCa = Bias-corrected accelerated. Bootstrapping based on 5,000 successful replicates.

Table S14. Logistic regression for anxiety symptoms among university students from Germany during the first wave of the COVID-19 pandemic

		95% *CI*					Wald Test			BCa 95% *CI*	
**Variable**	**Estimate**	**Lower bound**	**Upper bound**	**Standard Error**	**Odds Ratio**	*z*	**Wald Statistic**	*df*	*p*	**Lower bound**	**Upper bound**
Intercept	-22.372	-3221.57	3176.825	1632.273	1.922e -10	-0.014	1.879e -4	1	0.989	-41.319	-19.201
Gender (Women)	0.665	-0.958	2.288	0.828	1.945	0.804	0.646	1	0.422	-1.809	20.185
Place of Residence (City)	0.297	-1.228	1.821	0.778	1.345	0.381	0.146	1	0.703	-18.96	2.780
Level of Study (Master)	0.198	-1.073	1.469	0.649	1.219	0.305	0.093	1	0.760	-1.751	2.386
Exposure to COVID-19 (High)	17.571	-3181.63	3216.767	1632.273	4.277e +7	0.011	1.159e -4	1	0.991	-32.142	18.288
PIC Total (High)	0.920	-0.986	2.826	0.973	2.510	0.946	0.895	1	0.344	-2.457	4.307
PIC Qualifications (High)	1.908	-0.412	4.227	1.183	6.738	1.612	2.599	1	0.107	-43.958	19.059
PIC Economic Status (High)	0.753	-0.702	2.208	0.742	2.124	1.014	1.029	1	0.31	-3.297	2.926
PIC Relationships (High)	0.331	-1.127	1.789	0.744	1.392	0.445	0.198	1	0.657	-2.949	2.664
Physical Activity (Insufficient)	0.122	-1.197	1.442	0.673	1.130	0.182	0.033	1	0.856	-1.742	2.344
Physical Health (Worse)	-0.638	-2.144	0.868	0.768	0.528	-0.831	0.690	1	0.406	-2.930	2.302
Physical Health Comparative (Worse)	-0.699	-1.996	0.598	0.662	0.497	-1.056	1.116	1	0.291	-2.652	1.266

Note. *CI* = confidence interval; BCa = Bias-corrected accelerated. Bootstrapping based on 5,000 successful replicates.

Table S15. Logistic regression for anxiety symptoms among university students from Israel during the first wave of the COVID-19 pandemic

		95% *CI*					Wald Test			BCa 95% *CI*	
**Variable**	**Estimate**	**Lower bound**	**Upper bound**	**Standard Error**	**Odds Ratio**	*z*	**Wald Statistic**	*df*	*p*	**Lower bound**	**Upper bound**
Intercept	-3.066	-4.442	-1.69	0.702	0.047	-4.367	19.074	1	< .001	-4.355	-1.529
Gender (Women)	1.028	0.155	1.901	0.445	2.795	2.307	5.324	1	0.021	0.039	2.007
Place of Residence (City)	0.506	-0.383	1.503	0.481	1.751	1.164	1.355	1	0.244	-0.411	1.553
Level of Study (Master)	-	-	-	-	-	-	-	-	-	-	-
Exposure to COVID-19 (High)	0.648	-0.201	1.497	0.433	1.912	1.495	2.236	1	0.135	-0.351	1.556
PIC Total (High)	1.964	0.757	3.172	0.616	7.130	3.188	10.166	1	0.001	0.471	3.300
PIC Qualifications (High)	-0.118	-1.113	0.877	0.508	0.889	-0.233	0.054	1	0.816	-1.303	0.953
PIC Economic Status (High)	-0.858	-1.860	0.143	0.511	0.424	-1.680	2.821	1	0.093	-1.893	0.261
PIC Relationships (High)	0.548	-0.236	1.333	0.400	1.730	1.370	1.876	1	0.171	-0.350	1.384
Physical Activity (Insufficient)	-0.258	-0.996	0.479	0.376	0.772	-0.687	0.472	1	0.492	-1.104	0.631
Physical Health (Worse)	-	-	-	-	-	-	-	-	-	-	-
Physical Health Comparative (Worse)	0.264	-0.795	1.323	0.540	1.302	0.488	0.238	1	0.626	-0.801	1.256

Note. CI = confidence interval; BCa = Bias-corrected accelerated. Bootstrapping based on 5,000 successful replicates.

Table S16. Logistic regression for anxiety symptoms among university students from Poland during the first wave of the COVID-19 pandemic

		95% *CI*					Wald Test			BCa 95% *CI*	
**Variable**	**Estimate**	**Lower bound**	**Upper bound**	**Standard Error**	**Odds Ratio**	*z*	**Wald Statistic**	*df*	*p*	**Lower bound**	**Upper bound**
Intercept	-1.819	-2.795	-0.844	0.498	0.162	-3.656	13.365	1	< .001	-2.807	-0.691
Gender (Women)	-0.154	-1.080	0.772	0.472	0.857	-0.326	0.106	1	0.744	-1.104	0.902
Place of Residence (City)	0.216	-0.508	0.940	0.369	1.241	0.585	0.343	1	0.558	-0.609	0.991
Level of Study (Master)	-0.127	-0.771	0.517	0.329	0.880	-0.388	0.150	1	0.698	-0.860	0.578
Exposure to COVID-19 (High)	1.241	0.513	1.970	0.372	3.460	3.339	11.147	1	< .001	0.432	2.004
PIC Total (High)	0.539	-0.056	1.134	0.304	1.715	1.775	3.152	1	0.076	-0.163	1.155
PIC Qualifications (High)	-0.655	-1.271	-0.039	0.314	0.519	-2.085	4.346	1	0.037	-1.300	-0.001
PIC Economic Status (High)	0.062	-0.470	0.595	0.272	1.064	0.229	0.053	1	0.819	-0.515	0.632
PIC Relationships (High)	0.765	0.165	1.365	0.306	2.148	2.499	6.244	1	0.012	0.069	1.403
Physical Activity (Insufficient)	-0.479	-1.027	0.068	0.279	0.619	-1.716	2.944	1	0.086	-1.069	0.104
Physical Health (Worse)	1.978	0.297	3.659	0.858	7.228	2.306	5.317	1	0.021	-0.462	16.743
Physical Health Comparative (Worse)	0.819	-0.117	1.754	0.477	2.267	1.715	2.942	1	0.086	-0.273	1.842

Note. *CI* = confidence interval; BCa = Bias-corrected accelerated. Bootstrapping based on 5,000 successful replicates.

Table S17. Logistic regression for anxiety symptoms among university students from Russia during the first wave of the COVID-19 pandemic

		95% *CI*					Wald Test			BCa 95% *CI*	
**Variable**	**Estimate**	**Lower bound**	**Upper bound**	**Standard Error**	**Odds Ratio**	*z*	**Wald Statistic**	*df*	*p*	**Lower bound**	**Upper bound**
Intercept	-2.755	-3.765	-1.745	0.515	0.064	-5.345	28.569	1	< .001	-3.768	-1.589
Gender (Women)	0.565	-0.044	1.174	0.311	1.760	1.819	3.308	1	0.069	-0.092	1.173
Place of Residence (City)	0.091	-0.493	0.676	0.298	1.096	0.306	0.094	1	0.759	-0.572	0.718
Level of Study (Master)	0.707	-0.066	1.481	0.395	2.029	1.792	3.201	1	0.073	-0.195	1.539
Exposure to COVID-19 (High)	0.672	-0.01	1.354	0.348	1.958	1.931	3.727	1	0.054	-0.081	1.368
PIC Total (High)	-0.831	-1.737	0.075	0.462	0.436	-1.798	3.233	1	0.072	-1.853	0.194
PIC Qualifications (High)	0.781	0.059	1.503	0.368	2.183	2.119	4.491	1	0.034	-0.032	1.500
PIC Economic Status (High)	0.308	-0.367	0.982	0.344	1.361	0.895	0.801	1	0.371	-0.435	0.985
PIC Relationships (High)	1.145	0.446	1.843	0.356	3.141	3.213	10.321	1	0.001	0.318	1.973
Physical Activity (Insufficient)	-0.109	-0.686	0.468	0.294	0.897	-0.371	0.138	1	0.711	-0.756	0.503
Physical Health (Worse)	0.508	-0.314	1.330	0.419	1.662	1.211	1.466	1	0.226	-0.450	1.444
Physical Health Comparative (Worse)	0.529	-0.232	1.290	0.388	1.698	1.364	1.859	1	0.173	-0.279	1.430

Note. *CI* = confidence interval; BCa = Bias-corrected accelerated. Bootstrapping based on 5,000 successful replicates.

Table S18. Logistic regression for anxiety symptoms among university students from Slovenia during the first wave of the COVID-19 pandemic

		95% *CI*					Wald Test			BCa 95% *CI*	
**Variable**	**Estimate**	**Lower bound**	**Upper bound**	**Standard Error**	**Odds Ratio**	*z*	**Wald Statistic**	*df*	*p*	**Lower bound**	**Upper bound**
Intercept	-3.179	-4.669	-1.689	0.76	0.042	-4.182	17.493	1	< .001	-4.758	-1.337
Gender (Women)	0.462	-0.687	1.611	0.586	1.587	0.788	0.620	1	0.431	-0.94	1.753
Place of Residence (City)	-0.497	-1.35	0.356	0.435	0.608	-1.143	1.306	1	0.253	-1.527	0.487
Level of Study (Master)	-0.474	-1.318	0.370	0.431	0.623	-1.101	1.212	1	0.271	-1.388	0.558
Exposure to COVID-19 (High)	0.754	-0.127	1.636	0.450	2.126	1.677	2.812	1	0.094	-0.262	1.676
PIC Total (High)	0.968	-0.215	2.151	0.604	2.633	1.604	2.573	1	0.109	-0.551	2.351
PIC Qualifications (High)	0.865	-0.063	1.792	0.473	2.374	1.828	3.341	1	0.068	-0.357	1.872
PIC Economic Status (High)	-0.288	-1.191	0.616	0.461	0.750	-0.624	0.390	1	0.533	-1.325	0.744
PIC Relationships (High)	0.363	-0.569	1.295	0.476	1.437	0.762	0.581	1	0.446	-0.821	1.405
Physical Activity (Insufficient)	0.465	-0.306	1.235	0.393	1.592	1.183	1.398	1	0.237	-0.415	1.300
Physical Health (Worse)	0.598	-0.723	1.919	0.674	1.818	0.887	0.786	1	0.375	-0.941	1.927
Physical Health Comparative (Worse)	1.005	-0.110	2.119	0.569	2.731	1.767	3.123	1	0.077	-0.187	2.088

Note. *CI* = confidence interval; BCa = Bias-corrected accelerated. Bootstrapping based on 5,000 successful replicates.

Table S19. Logistic regression for anxiety symptoms among university students from Turkey during the first wave of the COVID-19 pandemic

		95% *CI*					Wald Test			BCa 95% *CI*	
**Variable**	**Estimate**	**Lower bound**	**Upper bound**	**Standard Error**	**Odds Ratio**	*z*	**Wald Statistic**	*df*	*p*	**Lower bound**	**Upper bound**
Intercept	-1.891	-3.021	-0.762	0.576	0.151	-3.283	10.776	1	0.001	-2.967	-0.717
Gender (Women)	0.656	0.162	1.151	0.252	1.928	2.602	6.771	1	0.009	0.111	1.175
Place of Residence (City)	-0.10	-0.647	0.447	0.279	0.905	-0.358	0.128	1	0.720	-0.685	0.471
Level of Study (Master)	-0.171	-1.036	0.693	0.441	0.842	-0.389	0.151	1	0.697	-1.071	0.745
Exposure to COVID-19 (High)	0.676	0.032	1.319	0.328	1.965	2.058	4.236	1	0.040	-0.007	1.309
PIC Total (High)	0.178	-0.697	1.053	0.446	1.195	0.399	0.159	1	0.690	-0.827	1.120
PIC Qualifications (High)	0.217	-0.416	0.850	0.323	1.242	0.671	0.451	1	0.502	-0.507	0.929
PIC Economic Status (High)	0.551	-0.044	1.146	0.304	1.734	1.813	3.289	1	0.070	-0.092	1.199
PIC Relationships (High)	0.472	-0.409	1.352	0.449	1.603	1.050	1.103	1	0.294	-0.491	1.356
Physical Activity (Insufficient)	-0.357	-1.051	0.338	0.354	0.700	-1.006	1.013	1	0.314	-1.085	0.409
Physical Health (Worse)	0.746	-0.286	1.778	0.527	2.108	1.416	2.006	1	0.157	-0.689	1.976
Physical Health Comparative (Worse)	0.427	-0.670	1.524	0.560	1.532	0.762	0.581	1	0.446	-1.013	1.721

Note. *CI* = confidence interval; BCa = Bias-corrected accelerated. Bootstrapping based on 5,000 successful replicates.

Table S20. Logistic regression for anxiety symptoms among university students from Ukraine during the first wave of the COVID-19 pandemic

		95% *CI*					Wald Test			BCa 95% *CI*	
**Variable**	**Estimate**	**Lower bound**	**Upper bound**	**Standard Error**	**Odds Ratio**	*z*	**Wald Statistic**	*df*	*p*	**Lower bound**	**Upper bound**
Intercept	-3.868	-5.202	-2.533	0.681	0.021	-5.682	32.281	1	< .001	-5.517	-2.140
Gender (Women)	0.876	0.148	1.603	0.371	2.400	2.360	5.571	1	0.018	0.101	1.639
Place of Residence (City)	-0.166	-0.847	0.515	0.348	0.847	-0.477	0.228	1	0.633	-0.923	0.560
Level of Study (Master)	-0.058	-1.495	1.379	0.733	0.944	-0.079	0.006	1	0.937	-14.561	1.471
Exposure to COVID-19 (High)	1.162	0.140	2.184	0.521	3.196	2.229	4.967	1	0.026	0.000	2.460
PIC Total (High)	0.341	-0.585	1.267	0.472	1.407	0.722	0.522	1	0.470	-0.599	1.337
PIC Qualifications (High)	0.633	-0.153	1.419	0.401	1.883	1.578	2.490	1	0.115	-0.208	1.365
PIC Economic Status (High)	0.140	-0.558	0.839	0.357	1.151	0.393	0.155	1	0.694	-0.610	0.900
PIC Relationships (High)	0.815	0.037	1.593	0.397	2.258	2.052	4.212	1	0.040	-0.132	1.745
Physical Activity (Insufficient)	-0.57	-1.179	0.039	0.311	0.566	-1.833	3.361	1	0.067	-1.207	0.068
Physical Health (Worse)	1.148	-0.346	2.641	0.762	3.150	1.506	2.269	1	0.132	-1.213	2.939
Physical Health Comparative (Worse)	0.291	-1.156	1.739	0.739	1.338	0.394	0.156	1	0.693	-1.825	2.064

Note. *CI* = confidence interval; BCa = Bias-corrected accelerated. Bootstrapping based on 5,000 successful replicates.
